# Completeness of medication information in admission notes from emergency departments

**DOI:** 10.1186/s12913-023-10371-4

**Published:** 2023-12-16

**Authors:** Birgitte Zahl-Holmstad, Beate H. Garcia, Kristian Svendsen, Tine Johnsgård, Renata V. Holis, Eirik H. Ofstad, Torsten Risør, Elin C. Lehnbom, Torbjørn Wisløff, Macty Chan, Renate Elenjord

**Affiliations:** 1 Hospital Pharmacy of North Norway Trust, Postboks 6147, Langnes, Tromsø, 9291 Norway; 2 Department of Pharmacy, Faculty of Health Sciences, UiT The Arctic University of Norway, Postboks 6050, Langnes, Tromsø, 9037 Norway; 3 Department of Medicine, Nordland Hospital Trust, Parkveien 95, Bodø, 8005 Norway; 4 Department of Community Medicine, Faculty of Health Sciences, UiT The Arctic University of Norway, Postboks 6050, Langnes, Tromsø, 9037 Norway; 5 Department of Public Health, Faculty of Health and Medical Sciences, University of Copenhagen, Øster Farimagsgade 5, Copenhagen K, 1014 Denmark; 6 Department of Health and Caring Sciences, Faculty of Health and Life Sciences, Linnaeus University, Universitetsplatsen 1, Kalmar, 392 31 Sweden; 7 Health Services Research Unit, Akershus University Hospital, Sykehusveien 25, Nordbyhagen, Lørenskog, 1478 Norway

**Keywords:** Patient safety, Quality of health care, Medication information, Admission notes, Electronic health records, Medication systems, Hospital

## Abstract

**Background:**

Medication lists prepared in the emergency department (ED) form the basis for diagnosing and treating patients during hospitalization. Since incomplete medication information may lead to patient harm, it is crucial to obtain a correct and complete medication list at hospital admission. In this cross-sectional retrospective study we wanted to explore medication information completeness in admission notes from Norwegian EDs and investigate which factors were associated with level of completeness.

**Methods:**

Medication information was assessed for completeness by applying five evaluation criteria; generic name, formulation, dose, frequency, and indication for use. A medication completeness score in percent was calculated per medication, per admission note and per criterion. Quantile regression analysis was applied to investigate which variables were associated with medication information completeness.

**Results:**

Admission notes for patients admitted between October 2018 and September 2019 and using at least one medication were included. A total of 1,080 admission notes, containing 8,604 medication orders, were assessed. The individual medications had a mean medication completeness score of 88.1% (SD 16.4), while admission notes had a mean medication completeness score of 86.3% (SD 16.2). Over 90% of all individual medications had information about generic name, formulation, dose and frequency stated, while indication for use was only present in 60%. The use of an electronic tool to prepare medication information had a significantly strong positive association with completeness. Hospital visit within the last 30 days, the patient’s living situation, number of medications in use, and which hospital the patient was admitted to, were also associated with information completeness.

**Conclusions:**

Medication information completeness in admission notes was high, but potential for improvement regarding documentation of indication for use was identified. Applying an electronic tool when preparing admission notes in EDs seems crucial to safeguard completeness of medication information.

**Supplementary Information:**

The online version contains supplementary material available at 10.1186/s12913-023-10371-4.

## Background

Care transitions, defined as “the movement of patients between health care practitioners, settings, and home, as their conditions and care needs change” [1], involve significant risk for medication-related errors [2, 3]. Poor communication between care levels may contribute to detrimental, but preventable, adverse events among patients [2, 4]. Incorrect medication information in patient records at hospital admission is common and potential consequences include patient discomfort, patient harm and deterioration of the patient’s condition [3]. In addition, incomplete medication information in admission notes increases the risk of consequential medication errors, and incomplete medication information in discharge summaries [5], which can lead to patient harm and unplanned hospital readmissions [6, 7].

Up to 29% of visits to emergency departments (EDs) [8, 9] and up to 64% of hospital readmissions are related to medications [10]. Therefore, it is important that information about a patient’s medication use at hospital admission is complete. Medication lists prepared at the ED admission follow the patients throughout the hospitalization and form the basis for assessments and decisions regarding further treatment. Hence, it is essential to obtain a correct and comprehensive medication list at hospital admission. Referral notes often lack medication lists or information about medication use, which makes the task of preparing a complete medication list in the ED time consuming and challenging [11, 12]. Other sources for information about medication use have fortunately become available in Scandinavia in recent years, including the introduction of a shared electronic medication list with an overview of electronic prescriptions and pharmacy-dispensed medications [13–15]. Another measure to improve the quality of medication information transfer is the utilization of medication reconciliation (MedRec).

MedRec may reduce medication discrepancies [16], medication errors and adverse drug events [17]. As a consequence, the Norwegian Patient Safety Program has fronted the implementation of MedRec in Norwegian hospitals to increase the quality of the medication lists and ultimately improve patient outcomes [18]. In the Northern Norway Regional Health Authority, MedRec is the physician’s responsibility and must be carried out according to a written procedure within 24–48 h of admission [19]. In the EDs, medication lists are normally prepared by junior physicians in their first year of specialization and documented electronically in the patients´ admission notes. An electronic tool incorporated in the electronic health record system makes this process simpler, since it automatically supplies information about generic name, dose, and medication formulation when trade name is entered. In addition, the tool gives a reminder to select frequency and state the indication for use. Although using the electronic tool is a requirement according to hospital procedure, it is also possible to write the medication information as free text in the admission note.

It is well known that medication discrepancies pose a significant patient threat during care transitions [2, 3]. MedRec serves as an effective method to identify and address such discrepancies [16]. However, the level of completeness of medication information remains a different concern. Research regarding medication information completeness in patient records has focused mainly on discharge summaries [11, 20–24], but also on referral notes [11, 12]. In general, studies have found an information deficit in a large proportion of the patient records and that the level of completeness is insufficient [11, 12, 20–23, 25, 26]. When it comes to medication information completeness in admission notes, research is scarce. Still, it is important, since medication information obtained in the ED serves as the foundation for further treatment.

The aim of this study was to explore medication information completeness in admission notes from Norwegian EDs, and to investigate which factors were associated with the level of completeness.

## Methods

### Study design, setting, sample selection and data collection

This was a cross-sectional retrospective study using patient record data from three hospitals in Norway involved in the PharmED study [27]. We included admission notes for patients acutely admitted to the hospital through the EDs during a 12-month period; 1^st^ October 2018 – 30^th^ September 2019. We aimed to assess the medication information in 30 admission notes from each hospital for each month, corresponding to 4.5% of all admission notes in this period. All of the admissions in each month were assigned a unique number and the associated admission notes to be assessed were randomly selected from this list applying Research Randomizer©. We consecutively reviewed admission notes until we had reached 30 admission notes per hospital per month for patients using at least one medication. To determine the proportion of medication users in our population and compare with the non-medication users, we also collected information about the non-medication users we identified when reviewing the admission notes. From the admission notes, we collected data about the patient, including age, sex, living situation, hospital visits within the last 30 days, and medication use. We also gathered information about cause of admission, arrival day and time to the ED, the work experience of the physician who wrote the admission note, if MedRec had been conducted and if the electronic tool was used or not. The variable “cause of admission” was based on which department the patient was admitted to when leaving the ED; either the department of medicine or the surgical/orthopaedic department. The variable “arrival time ED” was divided into busy time and non-busy time based on when the admission notes were written. The time interval in which most of them were written were deemed as busy time and was set to 10:00–22:00 Monday-Sunday.

### Assessment of medication information completeness

To assess medication information completeness in the admission notes, we applied evaluation criteria developed for evaluation of discharge summaries described by the Norwegian Patient Safety Program [28]. We included five criteria valid for admission notes, and excluded four, valid only for discharge summaries, see Table 1. Data on whether MedRec was documented on admission or not, were collected and included as a factor potentially influencing completeness in our analyses.

**Table 1:** Evaluation criteria applied, based on the Norwegian Patient Safety Program’s criteria for discharge summaries [28]

**Criteria**	**Included or excluded**	**Number of criteria in this study**
1. Is mediation reconciliation on admission documented?	**Included**	Factor for analyses
2. Is medication reconciliation on discharge correct?	Excluded	-
3. Is the source for medication information stated?	Excluded	-
4. Are reasons for changes stated?	Excluded	-
5. Are generic names stated?	**Included**	1
6. Are formulations stated?	**Included**	2
7. Are doses stated?	**Included**	3
8. Are frequencies stated?	**Included**	4
9. Are indications for use stated?	**Included**	5
10. Are categories stated?^a^	Excluded	-

^a^Refers to ICSD codes to be inserted in the discharge summary with each medication

*I* Initiated, *C* Changed, *S* Short course, *D* Discontinued

The five criteria were applied to each medication in the admission notes and received a score of 1 when the information was stated and a score of 0 if it was not. Hence, the total score a medication could receive was 5, if all criteria were applicable. Criteria could be inapplicable if the information was not possible to add in the admission note. An example of this would be criterion 1 (generic names stated) and criterion 3 (doses stated) for an intravenous nutrition additive that contained over 15 substances.

### Outcome measures

We calculated the *medication completeness score per medication* in *percent* by summing up the score of each criterion and dividing by the number of applicable criteria, multiplying with 100%.

We calculated the *medication completeness score per admission note* in *percent* by summing up the total score of all medications in the admission note and dividing by the number of medications in the admission note, multiplying with 100%.

We calculated the *medication completeness score per criterion* in *percent* by summing up the total number of medications having information about the criterion stated, dividing by the number of medications that had the respective criterion applicable, multiplying with 100%.

### Data management and analyses

Microsoft® Excel 365, STATA® 16.1 and IBM® SPSS Statistics 29 for Windows were used for data management and analyses. Continuous variables are presented with means and standard deviations (SDs), or median and interquartile ranges (IQRs) if not normally distributed. Categorical variables are presented as frequencies and percentages.

A quantile regression analysis was applied to investigate whether variables were associated with the outcome *mean medication completeness score of admission notes*. Quantile regression analysis was chosen since the assumptions of a regular multiple linear regression were not met, especially the assumption of a linear relationship between variables and outcome [29]. This type of regression allowed us to study the effect of variables at different levels of completeness, from the 10^th^ percentile admission notes with a low score of medication completeness to the 75^th^ percentile admission notes with a high score. Hence, we modelled the associations to the distribution of the outcome, rather than the mean [29]. We conducted the regression with four quantiles; 10^th^ percentile, 25^th^ percentile, 50^th^ percentile and 75^th^ percentile, as recommended by Staffa et al. for exploratory studies [29]. It was not possible to include the 90^th^ percentile, since the outcome (mean medication completeness score) in this quantile was 100%. The included variables in the regression analysis were selected based on factors that could plausibly be associated with the outcome. These factors were number of medications in use, age, sex, living situation, hospital visit last 30 days, cause of admission, arrival day ED, arrival time ED, hospital, MedRec conducted, applying the electronic tool, and experience of the physician.

A chi-squared-test was used to investigate the difference in medication completeness score per criterion in admission notes where the electronic tool had been applied versus the admission notes with free text.

The significance level in all analyses was set to *p* < 0.05.

## Results

### Demographics of study sample

**Fig. 1:**
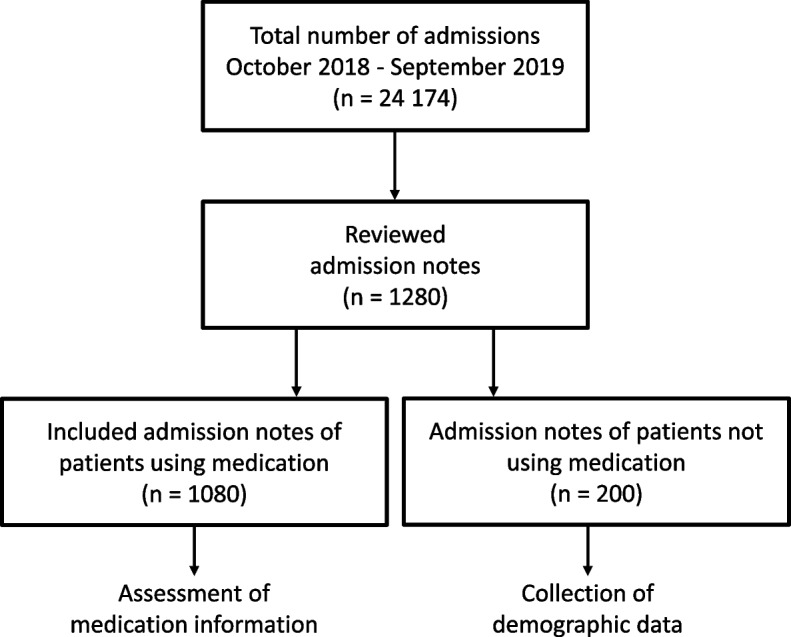
Flow chart showing admission notes included and assessed for medication information completeness

In total, we reviewed 1,280 admission notes; 1,080 (84.4%) were written for medication users, while 200 (15.6%) were written for non-medication users, see Fig. 1.

Medication users were between two and 101 years of age (median 72 years), and males represented 51.3% of them. Among the medication users, 20.2% had a previous hospitalization within the last 30 days, and 68.0% were admitted due to a medical cause, the rest due to a surgical cause. The non-medication user population was younger (median age 35 years), consisted of more males (58.5%), had fewer earlier hospitalizations the last 30 days (3.5%) and the majority was admitted due to a surgical cause (54.5%). Among the medication users, the median number of medications in use was seven for all medications, five for regular medications and two for as needed medications. See Table 2 for further details regarding demographics of the study sample.

**Table 2:** Demographics of the study sample

	**Medication user** (*n* = 1080)	**Non-medication user** (*n* = 200)
**Age**		
Median (Interquartile range)	72 (22)	35 (40)
Min/max	2–101	0–91
**Sex** (%)		
Male	554 (51.3)	117 (58.5)
**Living situation** (%)		
Home	911 (84.4)	186 (93.0)
Institution	112 (10.4)	3 (1.5)
Unknown	57 (5.3)	11 (5.5)
**Hospital visit last 30 days** (%)		
Yes	218 (20.2)	7 (3.5)
**Cause of admission** (%)		
Medical	734 (68.0)	91 (45.5)
Surgical	346 (32.0)	109 (54.5)
**Arrival day Emergency department** (%)		
Weekday	828 (76.7)	147 (73.5)
Weekend	252 (23.3)	53 (26.5)
**Arrival time Emergency department** (%)		
Busy time (10:00–22:00)	803 (74.4)	141 (71.0)
**Medication reconciliation documented** (%)		
Yes	865 (80.1)	74 (37.0)
**Medication use**		
**All medications**		
Median (Interquartile range)	7 (7)	-
Min/max	1–29	-
**Regular medications**		
Median (Interquartile range)	5 (6)	-
Min/max	0–21	-
**As needed medications**		
Median (Interquartile range)	2 (3)	-
Min/Max	0–14	-
**Preparation of medication information** (%)		
Electronic tool used	878 (81.3)	-
Free text	202 (18.7)	-

A total of 92.2% of the admission notes were written by junior physicians, while 0.9%, 5.9% and 0.8% were written by senior physicians, medical students, and physicians with unknown level of experience, respectively. The electronic tool was used to prepare the medication information in 81.3% of the admission notes, while physicians used free text in 18.7% of the notes.

### Medication information completeness

The admission notes contained a total of 8,604 medication orders; 5,863 of them were used regularly while 2,741 were used as needed. The individual medications achieved a mean medication completeness score of 88.1% (SD 16.4), where 56.2% of the individual medications achieved a score of 100% and 0.2% a score of 0%.

**Fig. 2:**
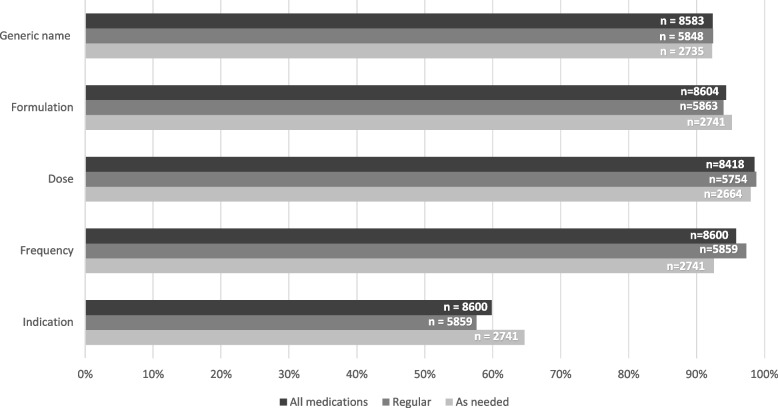
Medication completeness score per criterion (%). *n* = number of applicable criteria

Concerning each single criterion; generic name (criterion 1), formulation (criterion 2), dose (criterion 3) and frequency (criterion 4) all achieved high scores, with information present in 92.4%, 94.4%, 98.5%, and 95.8% of all medications, respectively. Indication for use (criterion 5) was only present in 59.9% of all the medications in the admission notes. For details regarding regular and as needed medications, see Fig. 2. Generic name was not applicable for 21 of the medications, dose was not applicable for 186 of the medications, and frequency and indication were not applicable for four medications each. The medications that most often were assessed “not applicable” were medications with multiple active substances, including vaccines, vitamins, and intravenous nutrition additives.

**Fig. 3:**
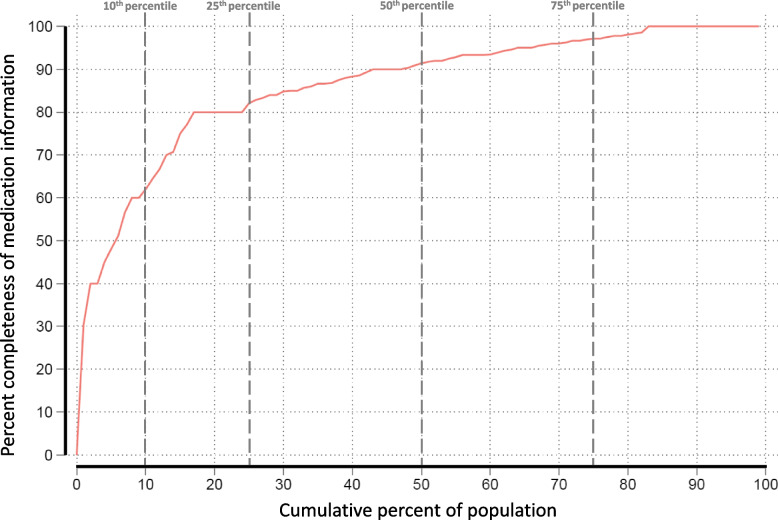
Medication completeness score (%) of admission notes (*n* = 1080), showed as cumulative percent of population

The mean medication completeness score of admission notes was 86.3% (SD 16.2). As Fig. 3 shows, the medication completeness score was high in the majority of admission notes, with a median of 91.4%. The 10^th^, 25^th^ and 75^th^ percentiles were 62.0%, 82.1%, and 97.1%, respectively. The proportion of admission notes achieving a full score for all medications was 17.4%.

### Factors influencing medication information completeness

The quantile regression analysis is shown in Table 3 and the descriptive statistics of the different quantiles (10^th^, 25^th^, 50^th^ and 75^th^ percentiles) according to *mean medication completeness score of admission notes* can be found in Additional file 1. The plot of the regression can be found in Additional file 2.

**Table 3:** Quantile regression showing association between mean medication completeness score of admissions notes (%) and variables

**Variable**	**Q1 (Quantile 1 – 10**^**th**^** percentile)**			**Q2 (Quantile 2 – 25**^**th**^** percentile)**			**Q3 (Quantile 3 – 50**^**th**^** percentile)**			**Q4 (Quantile 4 – 75**^**th**^** percentile)**		
	**Coefficient**	**(95% CI)**	***p* value**	**Coefficient**	**(95% CI)**	***p* value**	**Coefficient**	**(95% CI)**	***p* value**	**Coefficient**	**(95% CI)**	***p* value**
**Number of medications**	0.296	(0.185, 0.406)	< 0.001*	0.226	(0.073, 0.378)	0.004*	0.030	(-0.086, 0.146)	0.610	-0.213	(-0.294, -0.132)	< 0.001*
**Age**	0.033	(-0.023, 0.089)	0.245	0.074	(0.038, 0.111)	< 0.001*	0.009	(-0.019, 0.037)	0.545	-0.002	(-0.021, 0.017)	0.836
**Sex**												
Male	Ref.	-	-	Ref.	-	-	Ref.	-	-	Ref.	-	-
Female	-1.121	(-2.412, 0.170)	0.089	-1.251	(-2.758, 0.257)	0.104	-0.690	(-2.173, 0.792)	0.361	-0.360	(-0.884, 0.164)	0.178
**Living situation**												
Home	Ref.	-	-	Ref.	-	-	Ref.	-	-	Ref.	-	-
Institution	-1.590	(-3.053, -0.127)	0.033*	-2.717	(-4.333, -1.101)	0.001*	-2.655	(-5.235, -0.074)	0.044*	-2.367	(-4.391, -0.344)	0.022*
Unknown	-2.244	(-8.663, 4.175)	0.493	-0.089	(-5.594, 5.416)	0.975	-0.971	(-4.127, 2.184)	0.546	-0.360	(-3.494, 2.774)	0.822
**Hospital visit last 30 days**												
No	Ref.	-	-	Ref.	-	-	Ref.	-	-	Ref.	-	-
Yes	3.098	(0.947, 5.250)	0.005*	2.612	(1.035, 4.188)	0.001*	1.448	(0.258, 2.639)	0.017*	0.391	(-0.402, 1.185)	0.333
**Cause of admission**												
Surgical	Ref.	-	-	Ref.	-	-	Ref.	-	-	Ref.	-	-
Medical	-0.841	(-2.933, 1.251)	0.430	-0.512	(-1.931, 0.906)	0.479	-0.127	(-1.265, 1.012)	0.827	-0.077	(-0.840, 0.686)	0.844
**Arrival day ED**^**a**^												
Weekday	Ref.	-	-	Ref.	-	-	Ref.	-	-	Ref.	-	-
Weekend	-0.056	(-2.806, 2.694)	0.968	-0.789	(-2.831, 1.253)	0.449	0.018	(-1.600, 1.636)	0.983	0.208	(-0.939, 1.355)	0.722
**Arrival time ED**^**a**^												
Non-busy	Ref.	-	-	Ref.	-	-	Ref.	-	-	Ref.	-	-
Busy	-0.041	(-2.308, 2.226)	0.972	-1.804	(-4.068, 0.460)	0.118	-0.814	(-2.077, 0.449)	0.206	-0.840	(-1.640, -0.041)	0.039*
**Hospital**												
Hospital A	Ref.	-	-	Ref.	-	-	Ref.	-	-	Ref.	-	-
Hospital B	-2.143	(-3.773, -0.512)	0.010*	-5.110	(-6.992, -3.228)	< 0.001*	-3.678	(-4.610, -2.747)	< 0.001*	-1.665	(-3.064, -0.267)	0.020*
Hospital C	-0.511	(-3.158, 2.137)	0.705	-0.681	(-3.196, 1.835)	0.596	0.168	(-1.539, 1.876)	0.847	0.137	(-0.559, 0.832)	0.700
**Medication reconciliation**												
No				Ref.	-	-	Ref.	-	-	Ref.	-	-
Yes	1.006	(-1.817, 3.830)	0.485	1.374	(0.417, 2.332)	0.005*	1.552	(0.297, 2.806)	0.015*	0.854	(-0.569, 2.276)	0.239
**Free text or electronic tool**												
Free text	Ref.	-	-	Ref.	-	-	Ref.	-	-	Ref.	-	-
Electronic tool	41.284	(36.836, 45.733)	< 0.001*	39.541	(36.675, 42.407)	< 0.001*	33.131	(30.411, 35.851)	< 0.001*	24.837	(19.762, 29.912)	< 0.001*
**Experience of physician**												
Junior physician	Ref.	-	-	Ref.	-	-	Ref.	-	-	Ref.	-	-
Medical student	1.649	(-2.081, 5.379)	0.386	-0.143	(-2.479, 2.192)	0.904	1.483	(-1.465, 4.431)	0.324	0.583	(-0.319, 1.486)	0.205
Senior physician	0.172	(-4.622, 4.967)	0.944	-5.635	(-16.325, 5.055)	0.301	-11.894	(-31.340, 7.552)	0.230	0.595	(-17.744, 18.934)	0.949
Unknown	-10.033	(-29.970, 9.904)	0.324	1.042	(-13.678, 15.762)	0.890	3.202	(-15.430, 21.834)	0.736	0.437	(-20.436, 21.309)	0.967
**Constant**	37.486	(31.022, 43.951)	-	44.319	(39.275, 49.362)	-	60.410	(57.755, 63.066)	-	75.631	(70.187, 81.074)	-

^*^Statistically significant

^a^Emergency department

Our findings show that using the electronic tool to prepare medication information had the overall highest positive association with medication information completeness in admission notes. This association was greatest among the admission notes with the lowest completeness and decreased as the level of completeness increased. Hospital visit within the last 30 days had a positive association with medication information completeness within the admission notes with the lowest completeness. We identified that there was a negative association for completeness of information for patients living in an institution, compared to patients living at home or with an unknown living situation. In addition, we found a negative association for completeness of information for patients admitted to hospital B, compared to hospital A and hospital C. For the admission notes with the lowest completeness (Q1 and Q2), the number of medications had a positive association with completeness of information, with the completeness increasing with an increasing number of medications. For the admission notes with high level of completeness (Q4), we found a negative association between completeness and number of medications, with the increasing number of medications in use causing lower completeness.

**Fig. 4:**
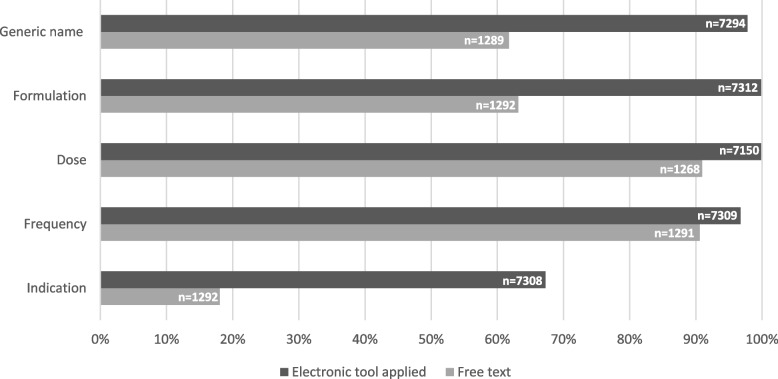
Medication completeness score per criterion (%), when applying the electronic tool versus free text. *n* = number of applicable criteria

Admission notes that had been prepared using the electronic tool had a higher mean medication completeness score (92.2%) compared to free text (60.4%). The criteria most vulnerable for achieving lower score with free text were generic name (criterion 1) and formulation (criterion 2), where about 40% of medications lacked information, see Fig. 4. Regarding indication for use (criterion 5), the criterion achieved a low score applying the digital tool (32.7% lacking information), but even lower with free text (82.0% lacking information). The differences in medication completeness score per criterion between the admission notes where the electronic tool was applied and the ones with free text were statistically significant (*p* = < 0.001).

## Discussion

This study shows a high medication information completeness in admission notes from three Norwegian EDs with a mean medication completeness score of 86.3%. More than half of the admission notes achieved a score higher than 90%. To our knowledge, no other studies have explored medication information completeness in admission notes. Consequently, we compare our findings with studies investigating discharge summaries and referral notes. These studies report different degrees of medication information completeness, but several of them found lower completeness compared to our findings. Hammad et al. found in their study from 2014 that 64.0% of discharge summaries contained information about all medications, including doses, frequencies, routes of administration, formulations and therapy duration [21]. A Norwegian study from 2017 found that the mean score for completeness of discharge summaries was 46.3% when assessing the following criteria: trade name, generic name, dosage, indication for use, medication changes accounted for, reasons for changes, categories stated behind each medication (as before, initiated, changed, short course, discontinued) and if source of medication information was stated [22]. In an Australian study from 2014, Lehnbom et al. compared medication information in paper and electronic discharge summaries and found completeness of 90.9% and 93.4%, respectively, when assessing the three criteria; dose, route and frequency [24]. Even though the mean medication completeness score of admission notes in our study was high, only 17.4% admission notes had a full score for all medications in the admission notes. This is in line with an Irish study from 2016 which found that overall compliance with all medication criteria (generic name, dose, frequency, duration of therapy, changes made, reason for changes and indications for newly started) were 18.9% of all discharge summaries [20].

We found that using the electronic tool had a strong positive association with medication information completeness and would theoretically increase the mean medication completeness score of admission notes by between 20–40%. On criteria level, we could see that use of the electronic tool provided significantly higher completeness for all the five criteria, and this is expected, since the electronic tool automatically supplies information and reminds the user to enter information. These results are in line with studies demonstrating that electronic discharge summaries had higher completeness than handwritten ones [21, 24] and computer-generated referral notes were more likely to include an accurate medication list compared to handwritten notes [12]. None of the admission notes included in this study were handwritten, but the ones prepared by free text can be compared to handwriting, since all information has to be typed in a free format by the physician. Transcription of medication information, whether it is electronic or handwritten, rather than automatically supplied, may lead to medication discrepancies and errors [30]. A study reporting that “free format elements” had lower compliance with information requirements in discharge summaries also supports our findings [20], in addition to a study that described handwritten discharge summary to be a strong predictor of poor medication information completeness [21]. Lehnbom et al. reported no large difference in completeness of information between paper and electronic discharge summaries, but the explanation was that the physician did not manually transcribe medication information; they attached a medication list directly printed from the electronic health record system to the paper discharge summary [24]. This strengthens the idea that use of electronic tools increases medication information completeness since manual transcription is avoided. It also voids any deciphering issues of poor handwriting.

When it comes to the number of medications in use per patient, we found that the association with completeness switched from positive to negative, as the completeness in the admission notes increased. These findings are in concordance with two studies presenting contradictory results; Hammad et al. found decreased completeness with increased number of medications, whereas Garcia et al. reported an increased completeness with increased number of medications [21, 22]. The explanation for this switch may be that physicians have more focus on getting medication information complete, the more medications a patient use. When a certain threshold of medications in use is reached, adding another medication to the medication list also will add an extra possibility to make an error, hence causes lower completeness. The regression analysis also revealed that *hospital visit within the last 30 days* had a positive association with medication information completeness. This is not surprising, since effort recently has been invested to collect information about medication use and creation of a complete medication list. However, what was surprising was that the factor *living in institution* had a negative association with completeness of information. One would think that patients in institutions, with health care professionals handling their medication, had a higher level of completeness than patients living at home and handling their own medication. The explanation may be that in Norwegian institutions, medication is prescribed and prepared in the local medicines storage rooms, which means that a shared electronic medication list does not exist and cannot be used as a source for information.

Our study showed that over 80% of patients admitted to the EDs are medication users, which emphasize the need to focus on medication safety and why completeness of information in electronic health records is important. The question which then occurs is; how can we get the medication information in electronic health records even more comprehensive and complete? Our findings support that if an electronic tool that can assist the physician exists, it is crucial to use it. The results also indicate that physicians still should prioritize giving extra attention to medication lists of patients using a large number of medications, in addition to patients living in institutions and patients that have not been hospitalized recently. We also identified that indication was the criteria most frequently missing, regardless of the electronic tool was used or not, and this is in line with other studies [20, 22]. It is a challenge for the ED physician to fill in information about indication when it is not stated in referral notes or patients’ electronic prescriptions. In order to do so, they would have to search through old documentation or even make assumptions. It is not easy to involve all patients either, since patients possess diverse views on their own medication use [31]. Physicians think MedRec is time-consuming detective work that cannot be prioritized in an ED setting [32], and when information about indication is not easily accessible, the physician will probably not focus on it. Consequently, completeness of information regarding indication of use should be an area to focus further on. Having this information stated in the admission note is important, both during the hospitalization, when the patient’s medication regime is being evaluated and modified, and at discharge, increasing the chance that indication is included in the discharge summary and communicated to the patient and the primary care providers. The identified differences in medication information completeness between hospitals imply that varying work flow, systems and culture may affect the focus on medication information completeness and medication safety. This highlights that measures on a system level should be considered implemented to ensure medication information completeness in electronic health records. Initiatives like prescriber education, MedRec and pharmacist partnership have been shown to have an positive effect on medication safety by reducing medications errors [33], and it is likely that such measures may also have an effect on medication information completeness. Implementing pharmacists in the ED reduce medication errors and improve quality of medication use [34] and MedRec performed by pharmacists reduce medication discrepancies in the ED [16]. This shows that pharmacists have a positive impact regarding medication safety in the ED and hopefully, future studies can investigate the impact of employing pharmacists in the ED on medication information completeness in admission notes.

### Strengths and limitations

The main strength of this study is the large number of randomly selected admission notes assessed. In addition, the criteria for assessing the medication information completeness were adopted from an already established set of evaluation criteria from a national patient safety program. When creating a score based on each of the five criteria, further analyses on this score implicitly implies equal weight to these criteria. Sometimes this can be problematic, but in this study, we consider the five criteria to be equally important. Even though we included data from three different hospitals, we found the same trends. It is therefore likely that our findings are generalizable to other EDs in Norway or elsewhere. We have studied the completeness of medication information, not the accuracy of it, which is a limitation. We assessed the medication information based on what was stated/documented in the admission note, we did not evaluate if the information was correct. There were also some variables missing from our data that may be associated with medication information completeness in the admission notes. Examples of this is degree of urgency/triage of the patient or who manages the patient’s medication when not in hospital.

## Conclusions

In this study we have identified that medication information completeness in admission notes in three Norwegian EDs is high. Still, there is potential for improvement, especially when it comes to documenting indication for use. We found that using an electronic tool integrated into the electronic health record system to prepare admission notes, significantly increased medication information completeness. These results highlight the importance of utilization of such electronic tools, when they are available, to safeguard medication information completeness. In addition, EDs should focus on getting medication information complete in patients living in an institution, that use a large number of medications, and have not recently been hospitalized.

### Supplementary Information

**Additional file 1.** Descriptive statistics of the different quantiles according to mean medication completeness score (%) of admission notes.

**Additional file 2.** Plot of the quantile regression.

## Data Availability

The datasets generated and analysed during this study are not openly available due to ethical and legal responsibility to protect our study sample’s identity. The retrospective data collection without patient consent was approved by the Regional Committee for Medical Research Ethics North Norway (REK), and we have not gained patient consent nor approval for publication of the data.

## Abbreviations

ED: Emergency department
MedRec: Medication reconciliation
Q: Quantile
