# Supplementary material for: Completeness of medication information in admission notes from emergency departments
Source: BMC Health Serv Res. 2023 Dec 16;23:1425. doi: 10.1186/s12913-023-10371-4 (PMC10724918; doi:10.1186/s12913-023-10371-4)
Supplement: Supplementary file 1 — Additional file 1. Descriptive statistics of the different quantiles according to mean medication completeness score (%) of admission notes. [file 12913_2023_10371_MOESM1_ESM.pdf]

Additional file 1: Descriptive statistics of the different quantiles according to mean medication completeness score (%) of admission notes

	Quantiles of Mean Medication Completeness Score (%)							
	**P1*** (n=107)		**P2**** (n=162)		**P3***** (n=534)		**P4***** (n=277)	
**Number of medications** (IQR)								
Median	4.0	(5)	5.0	(6)	9.0	(8)	7.0	(7)
Min/Max	1-19		1-23		2-29			1-24
**Age** (IQR)								
Median	61.0	(33)	70.0	(24)	74.0	(19)	70.0	(23)
Min/Max	2-99		10-97		11-101		13-95	
**Sex** (%)								
Male	47	(43.9)	81	(50.0)	271	(50.7)	155	(56.0)
**Living situation** (%)								
Home	90	(84.1)	128	(79.0)	435	(81.5)	258	(93.1)
Institution	6	(5.6)	20	(12.3)	79	(14.8)	7	(2.5)
Unknown	11	(10.3)	14	(8.6)	20	(3.7)	12	(4.3)
**Hospital visit last 30 days** (%)								
Yes	12	(11.2)	21	(13.0)	125	(23.4)	60	(21.7)
**Cause of admission** (%)								
Surgical	43	(40.2)	43	(26.5)	171	(32.0)	89	(32.1)
Medical	64	(59.8)	119	(73.5)	363	(68.0)	188	(67.9)
**Arrival day ED** (%)								
Weekday	76	(71.0)	120	(74.1)	416	(77.9)	216	(78.0)
Weekend	31	(29.0)	42	(25.9)	118	(22.1)	61	(22,0)
**Arrival time ED** (%)								
Non-busy								
Busy	78	(72.9)	120	(74.1)	411	(77.0)	194	(70.0)
**Hospital** (%)								
Hospital A	26	(24.3)	36	(22.2)	182	(34.1)	116	(41.9)
Hospital B	2	(1.9)	45	(27.8)	236	(44.2)	77	(27.8)
Hospital C	79	(73.8)	81	(50.0)	116	(21.7)	84	(30.3)
**Medication reconciliation** (%)								
Yes	46	(43.0)	123	(75.9)	455	(85.2)	241	(87.0)
**Free text or electronic tool** (%)								
Free text	105	(98.1)	76	(46.9)	19	(3.6)	2	(0.7)
Electronic tool	2	(1.9)	86	(53.1)	515	(96.4)	275	(99.3)
**Experience of physician** (%)								
Junior physician	98	(91.6)	146	(90.1)	500	(93.6)	253	(91.3)
Medical student	4	(3.7)	10	(6.2)	30	(5.6)	20	(7.2)
Senior physician	5	(4.7)	3	(1.9)	-		2	(0.7)
Unknown	-		3	(1.9)	4	(0.7)	2	(0.7)

* < 10th percentile with mean medication completeness score less than 62.0%
** 10th-25th percentile with mean medication completeness score 62.0-82.1%
*** 25th-75th percentile with mean medication completeness score 82.1-97.1%
**** >75th percentile with mean medication completeness score over 97.1%
