# Supplementary figures and images for: Completeness of medication information in admission notes from emergency departments
Source: BMC Health Serv Res. 2023 Dec 16;23:1425. doi: 10.1186/s12913-023-10371-4 (PMC10724918; doi:10.1186/s12913-023-10371-4)

## Additional file 2: Plot of the quantile regression.

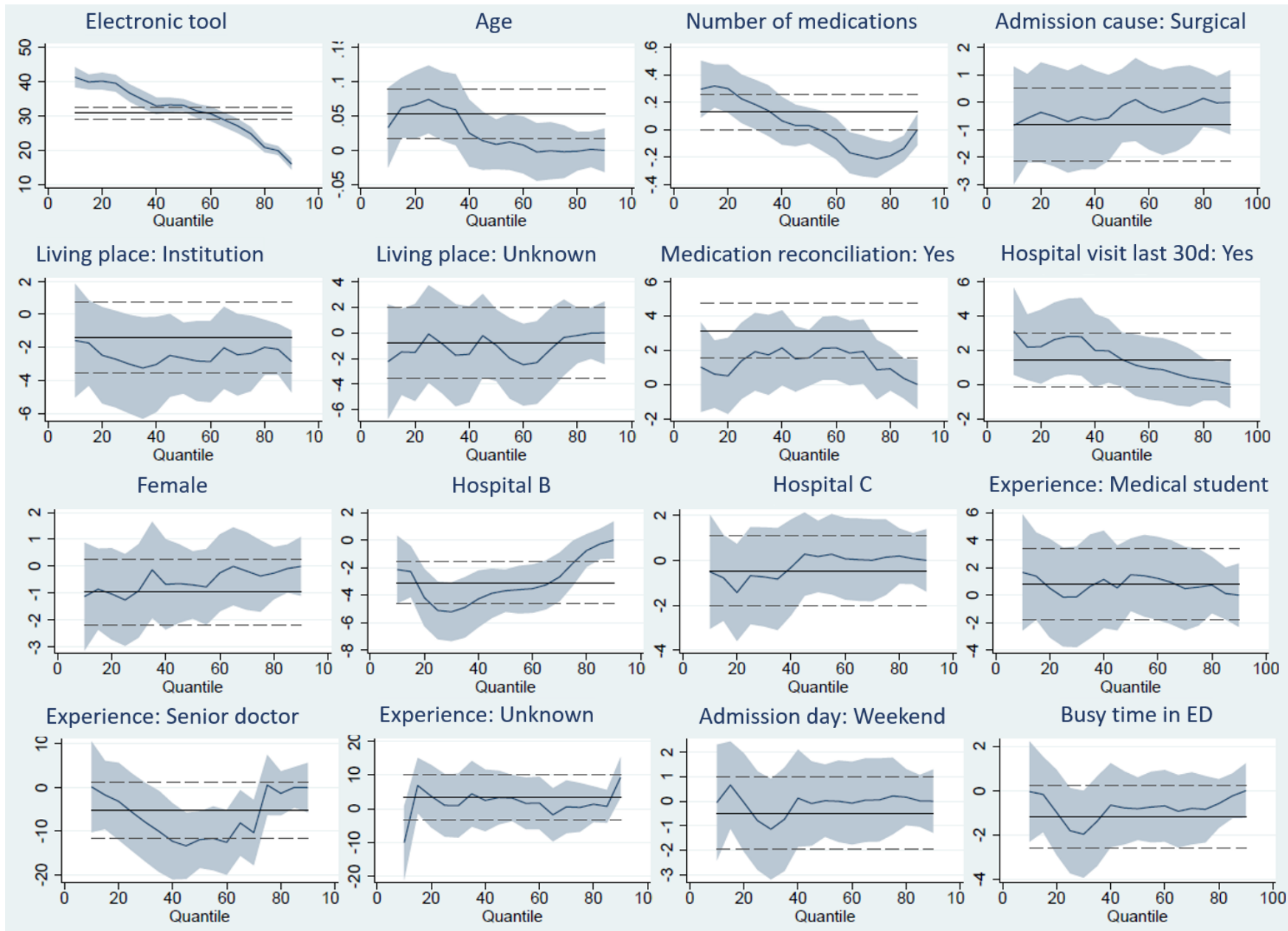

Note: Black line is regular linear regression

Supplement: Supplementary file 2 — Additional file 2. Plot of the quantile regression. [file 12913_2023_10371_MOESM2_ESM.pdf]
